# Fault diagnosis of nonlinear analog circuits using generalized frequency response function and LSSVM

**DOI:** 10.1371/journal.pone.0316151

**Published:** 2024-12-30

**Authors:** Jialiang Zhang, Yaowang Yang

**Affiliations:** 1 School of Electronic Information Engineering, Xi’an Technological University, Xi’an, Shaanxi, China; 2 Xi’an Special Equipment Inspection Institute, Xi’an, Shaanxi, China; Wilfrid Laurier University - Waterloo Campus: Wilfrid Laurier University, CANADA

## Abstract

A fault diagnosis method of nonlinear analog circuits is proposed that combines the generalized frequency response function (GFRF) and the simplified least squares support vector machine (LSSVM). In this study, the harmonic signal is used as an input to estimate the GFRFs. To improve the estimation accuracy, the GFRFs of an analog circuit are solved directly using time-domain data. The Fourier transform of the time-domain data is avoided. After obtaining the fault features, a multi-fault classifier is designed based on the LSSVM. In order to improve the training speed and reduces storage, a simplified LSSVM model is used to construct the classifier, and the conjugate gradient algorithm is used for training. The fault diagnosis simulation experiment is conducted on a biquad filter circuit to verify the proposed method. The experimental results show that the proposed method has high diagnostic accuracy and short training time.

## 1. Introduction

At present, analog circuits have been widely used in various electronic systems in the fields of automation, electronic information, communication, and robotics [1–4]. In electronic systems, most component failures occur in analog circuits, and if an analog circuit fails, the operation of the electronic system will be affected. With the increasing demand for the reliability of electronic systems, analog circuit fault diagnosis has become an important task in the field of circuit testing [5–8]. For the fault diagnosis of analog circuits, the wavelet packet transform and the generalized discriminant analysis have been used to obtain fault features, and a multi-kernel support vector machine (SVM) classifier has been used for fault recognition [9]. Kumar et al. [10] constructed a fuzzy classifier to diagnose faults in an analog circuit. Khanlari et al. [11] studied the diagnosis of multiple faults in analog circuits by using the kernel fuzzy C-means clustering method. The relationship between input and output of an analog circuit is very complex, and there is often a nonlinear mapping relationship between the circuit output and component parameter. In addition, the fault phenomenon of an analog circuit is very complex. If parameters of any component exceed the tolerance, the circuit is considered to be faulty. Therefore, it is of great significance to study the fault diagnosis of nonlinear analog circuits.

Analog circuit fault diagnosis includes two main parts: feature acquisition and fault recognition. Feature acquisition is very important for fault diagnosis performance of analog circuits. If the obtained features are not appropriate, the difference in the circuit performance under different fault modes can be reflected with difficulty, making it difficult for the diagnosis results to be satisfactory. Therefore, effective feature acquisition methods should be used to obtain fault feature variables. The Volterra series has been an effective mathematical tool for nonlinear system modeling and analysis [12–15]. In general, a nonlinear system can be described by a finite-order Volterra kernel. The Fourier transform of the Volterra kernel is called the generalized frequency response function (GFRF). The GFRF can be regarded as the extension of the frequency response function of a linear system to the nonlinear case and can describe the frequency domain characteristics of the nonlinear system [16–18]. Based on the cross-correlation strategy, a high-precision measurement method of a high-order frequency response function of a nonlinear system was proposed in [19]. For the GFRF estimation of a controlled plant in the closed-loop state, a calculation method based on a two-step strategy was proposed in [20]. These estimation methods require the Fourier transform of time-domain input and output data when solving the GFRF. A direct identification method of the GFRF based on the time-domain data, which takes a harmonic signal as an input and obtains the GFRF directly from the time-domain input and output data, was proposed in [21]. This method does not perform the Fourier transform operation on time-domain data, which reduces both the computational complexity and the amount of measurement data required for obtaining a solution.

After obtaining the feature variables, fault recognition is performed using these features, which represents another important task of analog circuit fault diagnosis. As a typical machine learning method, the SVM can effectively solve the problems of classification and regression and has good generalization ability [22–25]. At present, the SVM has been widely used in fault diagnosis [26–29]. In [30], feature variables were obtained by the coil current and contact travel, and an improved SVM was used to study the fault diagnosis of the high-voltage circuit breaker. Huang et al. [31] diagnosed the mechanical faults of a high-voltage circuit breaker using the genetic algorithm and SVM. Jegadeeshwaran et al. [32] conducted the fault diagnosis of an automotive hydraulic braking system by combining a decision tree and SVM. However, SVM training is needed to solve the quadratic programming problem, which has high computational complexity. Suykens et al. [33] proposed the LSSVM model by changing the risk function of the SVM. To reduce storage, in [34], an iterative algorithm based on a conjugate gradient was used for LSSVM training. Li et al. [35] proposed a simplified LSSVM model, which reduced the computational complexity.

In this paper, an analog circuit fault diagnosis method based on GFRF and simplified LSSVM is proposed. The generalized frequency response function is used to obtain the fault characteristic information, and a multi-classifier based on a LSSVM simplified model is constructed for fault recognition. First, a direct estimation method based on time-domain measurement data is used to obtain the GFRF of an analog circuit, which avoids the Fourier transform of input and output data. Then, the GFRF amplitude is used as fault feature. Finally, a simplified LSSVM is used to design a multi-fault classifier based on the conjugate gradient algorithm, which reduces the training complexity and required storage. The effectiveness of the proposed method is verified by the fault diagnosis simulation experiment on a biquad filter circuit.

## 2. Estimation of GFRF for nonlinear analog circuit

The Volterra series represents an extension of the Taylor series, which has been widely used to describe and analyze nonlinear systems. A nonlinear analog circuit is a typical nonlinear system, which has been widely used in various complex electromechanical equipment.

A single-input single-output nonlinear analog circuit with an input *u*(*t*) and an output *y*(*t*) can be expressed using the Volterra series as follows:

$$y(t)=\sum_{n=1}^{\infty }\int_{-\infty }^{\infty }\int_{-\infty }^{\infty }\cdots \int_{-\infty }^{\infty }h_{n}(\tau_{1},\tau_{2},\cdots ,\tau_{n})\prod_{i=1}^{n}u(t-\tau_{i})d\tau_{1}d\tau_{2}\cdots d\tau_{n}$$ (1)

where *h*_*n*_(*τ*_1_,*τ*_2_,⋯,*τ*_*n*_) denotes the *n*-order Volterra kernel of a nonlinear analog circuit.

The *n*-order output of an analog circuit is given by:

$$y_{n}(t)=\int_{-\infty }^{\infty }\int_{-\infty }^{\infty }\cdots \int_{-\infty }^{\infty }h_{n}(\tau_{1},\tau_{2},\cdots ,\tau_{n})\prod_{i=1}^{n}u(t-\tau_{i})d\tau_{1}d\tau_{2}\cdots d\tau_{n}$$ (2)

The Fourier transform of *h*_*n*_(*τ*_1_,⋯,*τ*_*n*_) is expressed as:

$$H_{n}(j\omega_{1},\cdots ,j\omega_{n})=\int_{-\infty }^{\infty }\cdots \int_{-\infty }^{\infty }h_{n}(\tau_{1},\cdots ,\tau_{n})e^{-j(\omega_{1}\tau_{1}+\cdots +\omega_{n}\tau_{n})}d\tau_{1}\cdots d\tau_{n}$$ (3)

where *H*_*n*_(j*ω*_1_,⋯,j*ω*_*n*_) denotes the *n*-order GFRF of an analog circuit.

Based on the GFRF, the output spectrum of a single-input single-output nonlinear analog circuit can be expressed as:

$$Y(j\omega )=\sum_{n=1}^{\infty }Y_{n}(j\omega )$$ (4)

$$\begin{array}{l} Y_{n}(j\omega )=\frac{1}{{\left(2\pi \right)}^{n-1}}\int_{-\infty }^{\infty }\int_{-\infty }^{\infty }\cdots \int_{-\infty }^{\infty }H_{n}(j\omega -j\omega_{1}-\cdots -j\omega_{n-1},j\omega_{1},\cdots ,j\omega_{n-1})\\ \;\cdot U(j\omega -j\omega_{1}-\cdots -j\omega_{n-1})U(j\omega_{1})\cdots U(j\omega_{n-1})d\omega_{1}d\omega_{2}\ldots d\omega_{n-1} \end{array}$$ (5)

where *Y*(j*ω*) is the output spectrum, *Y*_*n*_(j*ω*) is the *n*-order output spectrum, and *U*(∙) is the input spectrum.

The discrete form of the nonlinear analog circuit described by Eqs (1) and (2) can be expressed as:

$$y(k)=\sum_{n=1}^{\infty }y_{n}(k)$$ (6)

$$y_{n}(k)=\sum_{-\infty }^{\infty }\sum_{-\infty }^{\infty }\cdots \sum_{-\infty }^{\infty }h_{n}(i_{1},i_{2},\cdots ,i_{n})\prod_{m=1}^{n}u(k-i_{m})$$ (7)

In general, nonlinear analog circuits can be described by finite-order Volterra series. A single-input single-output nonlinear analog circuit described by the first three Volterra series can be expressed as follows:

$$y(k)=\sum_{n=1}^{3}\sum_{-\infty }^{\infty }\sum_{-\infty }^{\infty }\cdots \sum_{-\infty }^{\infty }h_{n}(i_{1},i_{2},\cdots ,i_{n})\prod_{m=1}^{n}u(k-i_{m}).$$ (8)

The discrete form of the output spectrum of the analog circuit can be expressed as:

$$\begin{array}{l} Y(j\omega )=H_{1}(j\omega )U(j\omega )+\sum_{\omega_{1}+\omega_{2}=\omega }H_{2}(j\omega_{1},j\omega_{2})U(j\omega_{1})U(j\omega_{2})\\ \;+\sum_{\omega_{3}+\omega_{4}+\omega_{5}=\omega }H_{3}(j\omega_{3},j\omega_{4},j\omega_{5})U(j\omega_{3})U(j\omega_{4})U(j\omega_{5}) \end{array}$$ (9)

where *H*_1_(*ω*), *H*_2_(j*ω*_1_,j*ω*_2_), and *H*_3_(j*ω*_3_,j*ω*_4_,j*ω*_5_) represent the first-, second-, and third-order GFRFs of the circuit, respectively.

By considering the measurement noise, the output of a nonlinear analog circuit can be expressed as follows:

$$\tilde{y}(k)=y(k)+e(k)$$ (10)

where, $\tilde{y}(k)$ is the measurement output, *y*(*k*) is the actual output, and *e*(*k*) is the zero mean Gaussian white noise.

The discrete form of the measured output spectrum of an analog circuit is expressed as:

$$\tilde{Y}(j\omega )=Y(j\omega )+\varepsilon (j\omega )$$ (11)

where *ε*(j*ω*) denotes the noise.

According to Eq (11), the GFRF of a nonlinear analog circuit can be estimated based on the least square principle. However, when using this method to obtain the GFRF, it is necessary to perform a Fourier transform on the time-domain measurement data, which has high computational complexity. In addition, the time-domain noise *e*(*k*) is the Gaussian white noise, but the frequency domain noise *ε*(j*ω*) may not be white noise, which may lead to a large deviation of GFRF estimation results.

To overcome these defects, Li et al. [21] proposed a simplified estimation method for the GFRF, which directly solves the GFRF using time-domain data. This estimation method is used to obtain the first three orders of the GFRF of a nonlinear analog circuit in this paper.

Using a harmonic signal *u*(*t*) = *A*cos*ωt* as an input, the discrete form of the measurement output of a single-input single-output nonlinear analog circuit can be expressed as follows:

y˜(k)=ARe{H1(jω)ejkTω}+2(A2)2Re{H2(jω,jω)ej2kTω}+2(A2)2Re{H2(jω,−jω)}+2(A2)3Re{H3(jω,jω,jω)ej3kTω}+6(A2)3Re{H3(jω,jω,−jω)ejkTω}+e(k) (12)

where Re represents the real part of a complex number, and *T* is the sampling period.

Assume *H*_1_(j*ω*),*H*_2_(j*ω*,j*ω*),*H*_2_(j*ω*−j*ω*),*H*_3_(j*ω*,j*ω*,j*ω*), and *H*_3_(j*ω*,j*ω*,j*ω*) are expressed as:

$$\left\{ \begin{array}{l} H_{1}(j\omega )=R_{1}(j\omega )+jI_{1}(j\omega )\\ H_{2}(j\omega ,j\omega )=R_{2}(j\omega ,j\omega )+jI_{2}(j\omega ,j\omega )\\ H_{2}(j\omega ,-j\omega )=R_{0}\\ H_{3}(j\omega ,j\omega ,j\omega )=R_{31}(j\omega ,j\omega ,j\omega )+jI_{31}(j\omega ,j\omega ,j\omega )\\ H_{3}(j\omega ,j\omega ,-j\omega )=R_{32}(j\omega ,j\omega ,-j\omega )+jI_{32}(j\omega ,j\omega ,-j\omega ) \end{array}\right.$$ (13)

where *R*_1_(j*ω*) and *I*_1_(j*ω*) are the real and imaginary parts of *H*_1_(j*ω*), respectively; *R*_2_(j*ω*,j*ω*) and *I*_2_(j*ω*,j*ω*) are the real and imaginary parts of *H*_2_(j*ω*,j*ω*), respectively; *R*_31_(j*ω*,j*ω*,j*ω*) and *I*_31_(j*ω*,j*ω*,j*ω*)are the real and imaginary parts of *H*_3_(j*ω*,j*ω*,j*ω*), respectively; *R*_32_(j*ω*,j*ω*,−j*ω*) and *I*_32_(j*ω*,j*ω*,−j*ω*) are the real and imaginary parts of *H*_3_(j*ω*,j*ω*,j*ω*), respectively; and *R*_0_ is a constant.

For simplicity, *R*_1_(j*ω*), *I*_1_(j*ω*), *R*_2_(j*ω*,j*ω*), *I*_2_(j*ω*,j*ω*), *R*_31_(j*ω*,j*ω*,j*ω*), *I*_31_(j*ω*,j*ω*,j*ω*), *R*_32_(j*ω*,j*ω*,−j*ω*), and *I*_32_(j*ω*,j*ω*,−j*ω*) are denoted by the abbreviated forms *R*_1_, *I*_1_, *R*_2_, *I*_2_ *R*_31_, *I*_31_, *R*_32_, and *I*_32_, respectively.

The term described by the first-order GFRF on the right side of Eq (12) can be expressed as:

ARe{H1(jω)ejkTω}=ARe{[R1+jI1][cos(kTω)+jsin(kTω)]}=AR1cos(kTω)−AI1sin(kTω) (14)

The terms described by the second-order GFRF on the right side of Eq (12) can be expressed as:

2(A2)2Re{H2(jω,jω)ej2kTω}=2(A2)2Re{[R2+jI2]⋅[cos(2kTω)+jsin(2kTω)]}=2(A2)2[R2cos(2kTω)−I2sin(2kTω)] (15)

2(A2)2Re{H2(jω,−jω)}=2(A2)2R0 (16)

The terms described by the third-order GFRF on the right side of Eq (12) can be expressed as:

2(A2)3Re{H3(jω,jω,jω)ej3kTω=2(A2)3Re{[R31+jI31][cos(3kTω)+jsin(3kTω)]}=2(A2)3[R31cos(3kTω)−I31sin(3kTω)] (17)

6(A2)3Re{H3(jω,jω,−jω)ejkTω=6(A2)3Re[R32+jI32][cos(kTω)+jsin(kTω)]=6(A2)3[R32cos(kTω)−I32sin(kTω)] (18)

According to Eqs (14)–(18), Eq (12) can be rewritten as follows:

$$\begin{array}{l} \tilde{y}(k)=[AR_{1}+6{\left(\frac{A}{2}\right)}^{3}R_{32})\cos(kT\omega )+[AI_{1}+6{\left(\frac{A}{2}\right)}^{3}I_{32})[-\sin(kT\omega )]\\ \;+2{\left(\frac{A}{2}\right)}^{2}R_{2}\cos(2kT\omega )-2{\left(\frac{A}{2}\right)}^{2}I_{2}\sin(2kT\omega )+2{\left(\frac{A}{2}\right)}^{2}R_{0}\\ \;+2{\left(\frac{A}{2}\right)}^{3}R_{31}\cos(3kT\omega )-2{\left(\frac{A}{2}\right)}^{3}I_{31}\sin(3kT\omega )+e(k) \end{array}$$ (19)

Define,

$$AR_{1}+6{\left(\frac{A}{2}\right)}^{3}R_{32}=R$$ (20)

$$AI_{1}+6{\left(\frac{A}{2}\right)}^{3}I_{32}=I$$ (21)

Let *k* = 1,2,⋯,*N*, then Eq (19) can be written in the form of a matrix, as follows:

Y=Xθ+E (22)

where $Y={\left[\tilde{y}(1),\cdots ,\tilde{y}(N)\right]}^{T}$, $\theta ={\left[R,I,R_{2},I_{2},R_{0},R_{31},I_{31}\right]}^{T}$, ***E*** = [*e*(1),⋯,*e*(*N*)]^*T*^, and

$$X=[\begin{array}{l} A\cos(T\omega ),\;-A\sin(T\omega ),\;2{\left(\frac{A}{2}\right)}^{2}\cos(2T\omega ),\;-2{\left(\frac{A}{2}\right)}^{2}\sin(2T\omega ),\;2{\left(\frac{A}{2}\right)}^{2},2{\left(\frac{A}{2}\right)}^{3}\cos(3T\omega ),\;-2{\left(\frac{A}{2}\right)}^{3}\sin(3T\omega )\\ A\cos(2T\omega ),\;-A\sin(2T\omega ),\;2{\left(\frac{A}{2}\right)}^{2}\cos(4T\omega ),\;-2{\left(\frac{A}{2}\right)}^{2}\sin(4T\omega ),\;2{\left(\frac{A}{2}\right)}^{2},2{\left(\frac{A}{2}\right)}^{3}\cos(6T\omega ),\;-2{\left(\frac{A}{2}\right)}^{3}\sin(6T\omega )\\ \;\vdots \\ A\cos(NT\omega ),\;-A\sin(NT\omega ),\;2{\left(\frac{A}{2}\right)}^{2}\cos(2NT\omega ),\;-2{\left(\frac{A}{2}\right)}^{2}\sin(2NT\omega ),\;2{\left(\frac{A}{2}\right)}^{2},2{\left(\frac{A}{2}\right)}^{3}\cos(3NT\omega ),\;-2{\left(\frac{A}{2}\right)}^{3}\sin(3NT\omega ) \end{array}].$$

According to the least square principle, we can get:

$$\hat{\theta }={\left(X^{T}X\right)}^{-1}X^{T}Y$$ (23)

where $\hat{\theta }={\left[\hat{R},\hat{I},{\hat{R}}_{2},{\hat{I}}_{2},{\hat{R}}_{0},{\hat{R}}_{31},{\hat{I}}_{31}\right]}^{T}$ denotes the estimated value of ***θ***.

To obtain *R*_1_, *I*_1_, *R*_32_, and *I*_32_, two harmonic signals with the same frequency but different amplitudes can be used to excite a nonlinear analog circuit.

Assume the input signals are *u*_1_(*t*) = *A*_1_ cos *ωt* and *u*_2_(*t*) = *A*_2_ cos *ωt*, according to Eqs (20) and (21), the following equation can be obtained:

$$\left\{ \begin{array}{l} A_{1}{\hat{R}}_{1}+6{\left(\frac{A_{1}}{2}\right)}^{3}{\hat{R}}_{32}={\hat{R}}^{\left(1\right)}\\ A_{1}{\hat{I}}_{1}+6{\left(\frac{A_{1}}{2}\right)}^{3}{\hat{I}}_{32}={\hat{I}}^{\left(1\right)}\\ A_{2}{\hat{R}}_{1}+6{\left(\frac{A_{2}}{2}\right)}^{3}{\hat{R}}_{32}={\hat{R}}^{\left(2\right)}\\ A_{2}{\hat{I}}_{1}+6{\left(\frac{A_{2}}{2}\right)}^{3}{\hat{I}}_{32}={\hat{I}}^{\left(2\right)} \end{array}\right.$$ (24)

By solving Eq (24), we can obtain:

$$\left\{ \begin{array}{l} {\hat{R}}_{1}=\frac{6{\left(A_{2}/2\right)}^{3}{\hat{R}}_{1}^{\prime }-6{\left(A_{1}/2\right)}^{3}{\hat{R}}_{2}^{\prime }}{A_{1}\cdot 6{\left(A_{2}/2\right)}^{3}-A_{2}\cdot 6{\left(A_{1}/2\right)}^{3}}\\ {\hat{I}}_{1}=\frac{6{\left(A_{2}/2\right)}^{3}{\hat{I}}_{1}^{\prime }-6{\left(A_{1}/2\right)}^{3}{\hat{I}}_{2}^{\prime }}{A_{1}\cdot 6{\left(A_{2}/2\right)}^{3}-A_{2}\cdot 6{\left(A_{1}/2\right)}^{3}}\\ {\hat{R}}_{32}=\frac{A_{2}{\hat{R}}_{1}^{\prime }-A_{1}{\hat{R}}_{2}^{\prime }}{A_{2}\cdot 6{\left(A_{1}/2\right)}^{3}-A_{1}\cdot 6{\left(A_{2}/2\right)}^{3}}\\ {\hat{I}}_{32}=\frac{A_{2}{\hat{I}}_{1}^{\prime }-A_{1}{\hat{I}}_{2}^{\prime }}{A_{2}\cdot 6{\left(A_{1}/2\right)}^{3}-A_{1}\cdot 6{\left(A_{2}/2\right)}^{3}} \end{array}\right.$$ (25)

The GFRF of a nonlinear system can reflect the essential characteristics of the system. Therefore, the feature information obtained by the GFRF can be effectively used for fault diagnosis of nonlinear analog circuits. The amplitudes reflect the main information of GFRFs. Therefore, the amplitudes of GFRFs are used as fault feature variables in this paper.

## 3. Multi-fault classifier based on simplified LSSVM

The SVM is a typical machine learning-based method that can effectively solve the classification problem. The SVM classifier maps the input vector to a high-dimensional feature space through a nonlinear transformation first, and then constructs an optimal classification hyperplane to classify the vector.

The SVM training requires solving a quadratic programming problem with high computational complexity. To improve the training speed, Suykens et al. [33] proposed the LSSVM, which is based on the SVM. In this paper, the LSSVM is used to design a multi-fault classifier for fault recognition of a nonlinear analog circuit.

Assume a training sample dataset $S_{1}:{\left\{(x_{i},y_{i})\right\}}_{i=1}^{M}$, where *x*_*i*_∈**R**^*n*^is the *i*th input pattern, *y*_*i*_∈{−1,1} is the *i*th output pattern, and *M* is the sample size.

The LSSVM classification problem can be defined as follows:

$$\begin{array}{l} \min\;J_{1}(w,b,\varepsilon )=\frac{1}{2}w^{Τ}w+\frac{1}{2}\gamma \sum_{i=1}^{M}\varepsilon_{i}^{2}\\ s.t.\;y_{i}[w^{Τ}\varphi (x_{i})+b]=1-\varepsilon_{i},\;i=1,2,\cdots ,M \end{array}$$ (26)

where ***w*** is the weight vector of the classification hyperplane, *b* is the classification threshold, *ε* = [*ε*_1_,⋯,*ε*_*M*_] is the slack vector, *γ*>0 is the penalty factor, and *φ*(∙) represents the nonlinear mapping operation.

The Lagrangian function can be constructed as follows:

$$L(w,b,\varepsilon ,\alpha )=\frac{1}{2}w^{Τ}w+\frac{1}{2}\gamma \sum_{i=1}^{M}\varepsilon_{i}^{2}-\sum_{i=1}^{M}\alpha_{i}\{y_{i}[w^{Τ}\varphi (x_{i})+b]-1+\varepsilon_{i}\}$$ (27)

where ***α*** = [*α*_1_,⋯,*α*_*M*_]^*T*^ denote Lagrange multiplier vectors.

Let the partial derivatives of the Lagrangian function *L*(***w***,*b*,***ε***,***α***) with respect to ***w***, *b*, *ε*_*i*_, and *α*_*i*_ be zeros, then,

$$\left\{ \begin{array}{l} \frac{\partial L}{\partial w}=0\Rightarrow w=\sum_{i=1}^{M}\alpha_{i}y_{i}\varphi (x_{i})\\ \frac{\partial L}{\partial b}=0\Rightarrow \sum_{i=1}^{M}\alpha_{i}y_{i}=0\\ \frac{\partial L}{\partial \varepsilon_{i}}=0\Rightarrow \alpha_{i}=C\varepsilon_{i}\\ \frac{\partial L}{\partial \alpha_{i}}=0\Rightarrow y_{i}[w^{Τ}\varphi (x_{i})+b]-1+\varepsilon_{i}=0 \end{array}\right.$$ (28)

According to Eq (28), the constrained optimization problem of the LSSVM described by Eq (26) can be transformed into a set of linear equations:

$$\left[\begin{array}{l} 0\;Y^{T}\\ Y\;H \end{array}]\cdot [\begin{array}{l} b\\ \alpha \end{array}]=[\begin{array}{l} 0\\ 1 \end{array}\right]$$ (29)

where ***Y*** = [*y*_1_,*y*_2_,⋯,*y*_*M*_]^*T*^; ***H*** = **Ω**+*γ*^−1^ ***I***, where **Ω** is an *M*-dimensional symmetric square matrix; Ω_*i*,*j*_ = *y*_*i*_*y*_*j*_*K*(*x*_*i*_,*x*_*j*_), where *K*( , ) is a kernel function; *I* is an *M*-dimensional identity matrix; ***α*** = [*α*_1_,*α*_2_,⋯,*α*_*M*_]^*T*^ is the Lagrange multiplier vector, and **1** = [1,1,⋯,1]^*T*^.

Choosing an appropriate kernel function is very important to ensure the performance of an LSSVM classifier. The commonly used kernel functions include polynomial function, Gaussian radial basis function, and sigmoid function. Because the Gaussian radial basis function has good classification performance, it is used to design a multi-fault classifier in this study, and it is given by:

$$K_{RBF}(x_{i},x_{j})=\exp[-\frac{{\left\|x_{i}-x_{j}\right\|}^{2}}{\sigma^{2}}]$$ (30)

where *σ* is the width of the radial basis function.

Obviously, the matrix on the left side of Eq (29) is an (*M*+1)-order square matrix. When the sample size *M* is large, the matrix inversion operation needs a large amount of memory. Therefore, to reduce the required storage, an iterative algorithm based on the conjugate gradient is used to train the LSSVM [34]. There are two *M*-variable linear equations to be solved when using the traditional LSSVM model and conjugate gradient algorithm to train an LSSVM binary classifier. To reduce the amount of calculation, Li et al. [35] proposed a simplified LSSVM model.

The above-presented LSSVM classifier is a binary classifier. For the classification of multiple patterns, a multi-classifier can be constructed by combining multiple binary classifiers. There are many types of faults in nonlinear analog circuits, so in this study, a one-against-one LSSVM multi-classifier is constructed for fault recognition. To improve the training speed of a multi-fault classifier and to reduce the storage required for calculation, this study uses a conjugate gradient algorithm to train the classifier based on the simplified LSSVM model.

Assume that for a multi-fault LSSVM classifier, the training sample set of the *k*th binary sub-classifier is denoted by *G*_*k*_: {***g***_*i*_,*y*_*i*_},*i* = 1,2,⋯,*M*_*k*_, where ***g***_*i*_∈**R**^*p*^ is the GFRF feature vector, *y*_*i*_∈{−1,1} is the category label, and *M*_*k*_ is the sample size. Then, the following set of linear equations can be obtained:

$$\left[\begin{array}{l} 0\;Y_{k}^{T}\\ Y_{k}\;H_{k} \end{array}]\cdot [\begin{array}{l} b_{k}\\ \alpha_{k} \end{array}]=[\begin{array}{l} 0\\ 1 \end{array}\right]$$ (31)

where $Y_{k}={\left[y_{1},y_{2}\cdots ,y_{M_{k}}\right]}^{T}$; $H_{k}=\Omega_{k}+c_{k}^{-1}\cdot I_{k}$; **Ω**_*k*_ is an *M*_k_-dimensional symmetric square matrix; Ω_*i*,*j*_ = *y*_*i*_*y*_*j*_*K*_*RBF*_(*x*_*i*_,*x*_*j*_); *I* is an *M*_k_-dimensional identity matrix; $\alpha_{k}=[\alpha_{1},\alpha_{2}\cdots ,\alpha_{M_{k}}]$ is the Lagrange multiplier vector, and **1** = [1,1,⋯,1]^*T*^.

The dual problem of an LSSVM binary classifier can be expressed as:

$$\begin{array}{l} \min\;J_{2}(\alpha_{k})=\frac{1}{2}\alpha_{k}^{T}H_{k}\alpha_{k}-\alpha_{k}^{T}\cdot 1\\ s.t.\;Y_{k}^{T}\alpha_{k}=0 \end{array}$$ (32)

The matrix ***H***_*k*_ can be expressed as:

$$H_{k}=[\begin{array}{l} H^{M_{k}-1},\;h_{k}\\ \;h_{k}^{T},\;H_{M_{k}M_{k}} \end{array}]$$ (33)

where $H^{M_{k}-1}$ is an (*M*_*k*_−1)×(*M*_*k*_−1) principal square sub-matrix of ***H***_*k*_; ***h***_*k*_ is an (*M*_*k*_−1)-dimensional vector after the last element is removed from the *M*_*k*_th vector of ***H***_*k*_; and $H_{M_{k}M_{k}}$ is the element in the row *M*_*k*_ and column *M*_*k*_ of ***H***_*k*_.

Define

$$Y_{k}^{\left(M_{k}-1\right)}={\left[y_{1},y_{2},\cdots ,y_{M_{k}-1}\right]}^{T}$$ (34)

$$\alpha_{k}^{\left(M_{k}-1\right)}={\left[\alpha_{1},\alpha_{2},\cdots ,\alpha_{M_{k}-1}\right]}^{T}$$ (35)

By substituting Eqs (38)–(40) into Eq (37), an unconstrained quadratic minimization problem can be defined as follows:

$$\min\;J_{3}(\alpha_{k})=\frac{1}{2}{\left(\alpha_{k}^{\left(M_{k}-1\right)}\right)}^{T}{\tilde{H}}_{k}\alpha_{k}^{\left(M_{k}-1\right)}-{\left(1-y_{M_{k}}Y_{k}^{\left(M_{k}-1\right)}\right)}^{T}\alpha_{k}^{\left(M_{k}-1\right)}$$ (36)

where ${\tilde{H}}_{k}=H^{M_{k}-1}-y_{M_{k}}Y_{k}^{\left(M_{k}-1\right)}h_{k}^{T}-y_{M_{k}}h_{k}{\left(Y_{k}^{\left(M_{k}-1\right)}\right)}^{T}+H_{M_{k}M_{k}}Y_{k}^{\left(M_{k}-1\right)}{\left(Y_{k}^{\left(M_{k}-1\right)}\right)}^{T}$ is a positive definite symmetric matrix.

According to Eqs (32)–(36), the simplified model of the LSSVM classifier is given by:

$$\left\{ \begin{array}{l} {\tilde{H}}_{k}\alpha_{k}^{\left(M_{k}-1\right)}=1-y_{M_{k}}Y_{k}^{\left(M_{k}-1\right)}\\ \alpha_{M_{k}}=-y_{M_{k}}(Y_{k}^{\left(M_{k}-1\right)}{)}^{T}\alpha_{k}^{\left(M_{k}-1\right)}\\ b_{k}=y_{M_{k}}-y_{M_{k}}{\left(H_{k}\alpha_{k}\right)}_{M_{k}} \end{array}\right.$$ (37)

where ${\left(H_{k}\alpha_{k}\right)}_{M_{k}}$ is the *M*_*k*_th element of *H*_*k*_***α***_*k*_.

**Fig 1:**
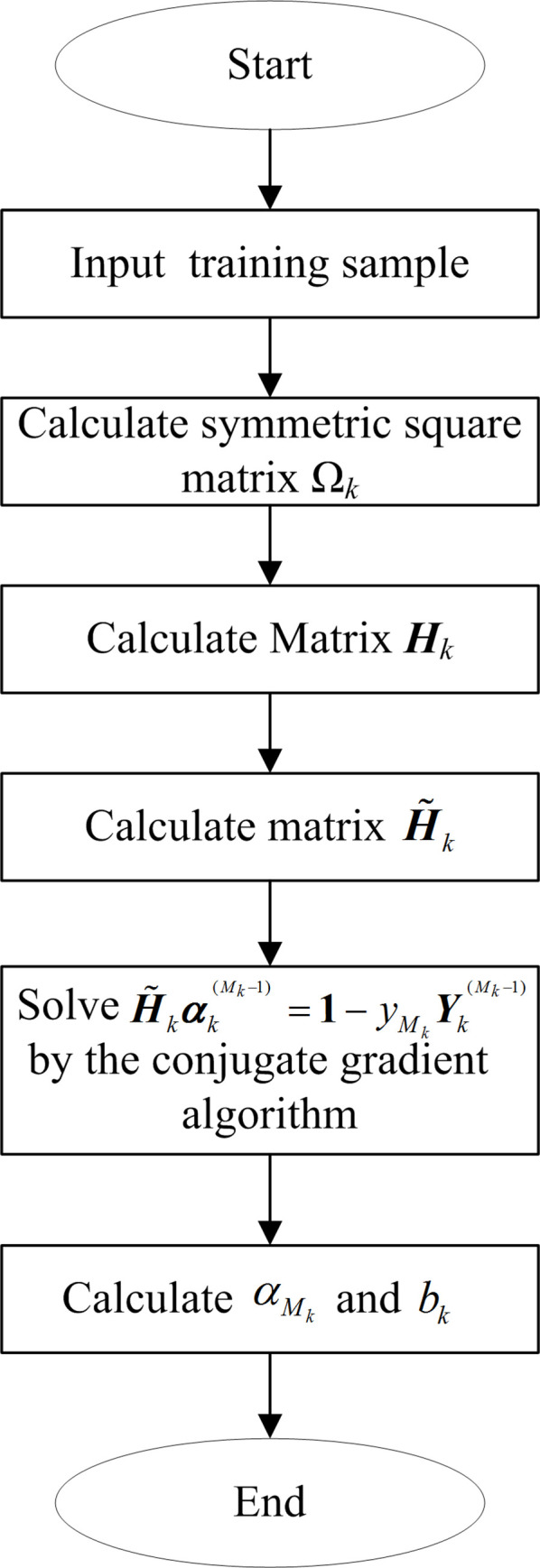
The training flowchart of the *k*th binary LSSVM sub-classifier.

The training flowchart of the *k*th binary LSSVM sub-classifier is shown in Fig 1. When training a sub-classifier of a multi-fault classifier of a nonlinear analog circuit according to the simplified LSSVM classifier model, the matrices **Ω**_*k*_, ***H***_*k*_ and ${\tilde{H}}_{k}$ are caculated firstly. Then, the conjugate gradient algorithm is used to solve the (*M*-1)-variable linear equations ${\tilde{H}}_{k}\alpha_{k}^{\left(M_{k}-1\right)}=1-y_{M_{k}}Y_{k}^{\left(M_{k}-1\right)}$ and to obtain the Lagrange multiplier vector $\alpha_{k}^{\left(M_{k}-1\right)}$. Finally, the Lagrangian multiplier $\alpha_{M_{k}}$ and the classification threshold *b*_*k*_ can be calculated by using $\alpha_{M_{k}}=-y_{M_{k}}{\left(Y_{k}^{\left(M_{k}-1\right)}\right)}^{T}\alpha_{k}^{\left(M_{k}-1\right)}$ and $b_{k}=y_{M_{k}}-y_{M_{k}}{\left(H_{k}\alpha_{k}\right)}_{M_{k}}$.

When using the simplified LSSVM model to train the *k*th LSSVM binary classifier, there is one (*M*-1)-variable linear equation ${\tilde{H}}_{k}\alpha_{k}^{\left(M_{k}-1\right)}=1-y_{M_{k}}Y_{k}^{\left(M_{k}-1\right)}$ need to be solved. When using the traditional LSSVM model to train the *k*th LSSVM binary classifier of the multi-fault classifier for a nonlinear analog circuit, there are two *M*-variable linear equations ***H***_*k*_***η***_*k*_ = ***Y***_*k*_ and ***H***_*k*_***υ***_*k*_ = **1** need to be solved. Therefore, the computational complexity of the simplified LSSVM model is significantly reduced.

**Fig 2:**
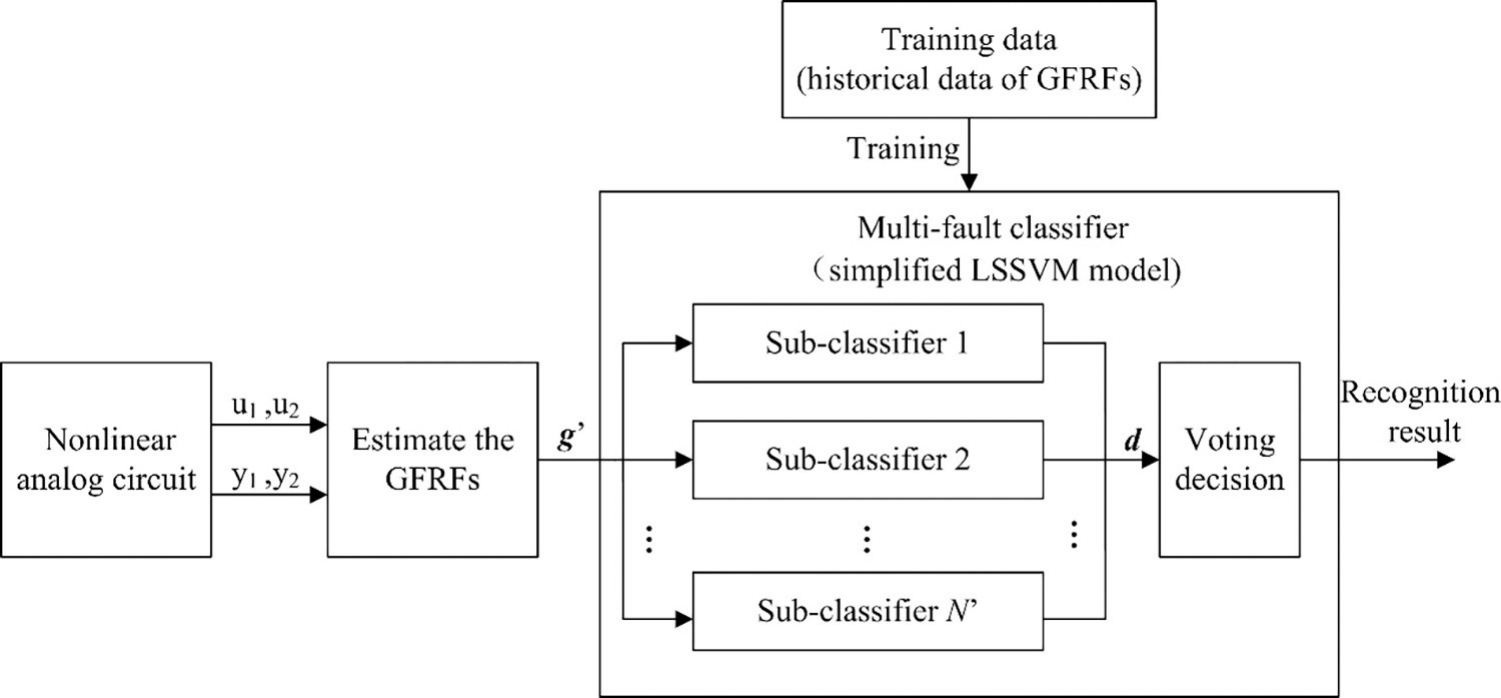
The schematic diagram of fault diagnosis process.

The schematic diagram of fault diagnosis process is shown in Fig 2.

The steps of the fault diagnosis for a nonlinear analog circuit based on the GFRF and simplified LSSVM are as follows:

**Step 1:** Select two harmonic signals with the same frequency but different amplitudes to excite a nonlinear analog circuit and obtain the input and output data;

**Step 2:** Estimate the GFRFs of the circuit system according to Eqs (23) and (25);

**Step 3:** Use the amplitudes of the GFRFs of the nonlinear analog circuit as a fault feature vector ***g*′**;

**Step 4:** Send the feature vector ***g*′** to each binary sub-classifier of the LSSVM multi-fault classifier and obtain the decision vector ***d***;

**Step 5:** Make the voting decision according to vector ***d*** and obtain the fault recognition result.

## 4. Fault diagnosis experiment and analysis

**Fig 3:**
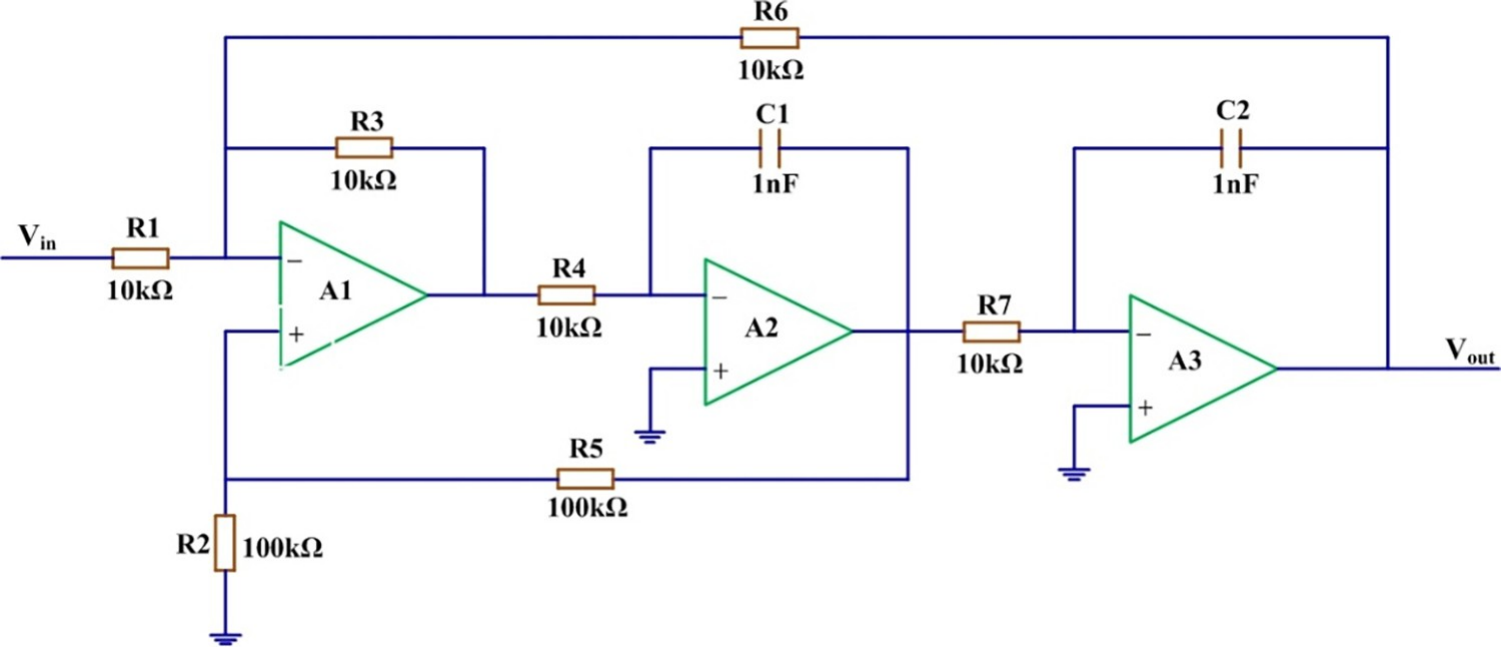
The biquad filter circuit.

To verify the effectiveness of the proposed fault diagnosis method for nonlinear analog circuits, a biquad filter is used in the simulation experiment. Fig 3 shows a biquad filter circuit, where, *R*_1_,*R*_2_,⋯,*R*_7_ denote resistances, *C*_1_ and *C*_2_ are capacitances, *V*_*in*_ is input voltage, and *V*_*out*_ is output voltages, respectively.

The fault information on the biquad filter circuit, including nine fault modes, is given in Table 1. In the experiment, it is assumed that in the case of a failure of circuit elements, the element parameters either increased or decreased by 30%.

**Table 1:** The fault description of the biquad filter circuit used in experiments.

Fault code	Fault description	Nominal value	Fault value
F0	NF	-	-
F1	R3↑	10 kΩ	13 kΩ
F2	R3↓	10 kΩ	7 kΩ
F3	R5↑	100 kΩ	130 kΩ
F4	R5↓	100 kΩ	70 kΩ
F5	C1↑	1 nF	1.3 nF
F6	C1↓	1 nF	0.7 nF
F7	C2↑	1 nF	1.3 nF
F8	C2↓	1 nF	0.7 nF

**Fig 4:**
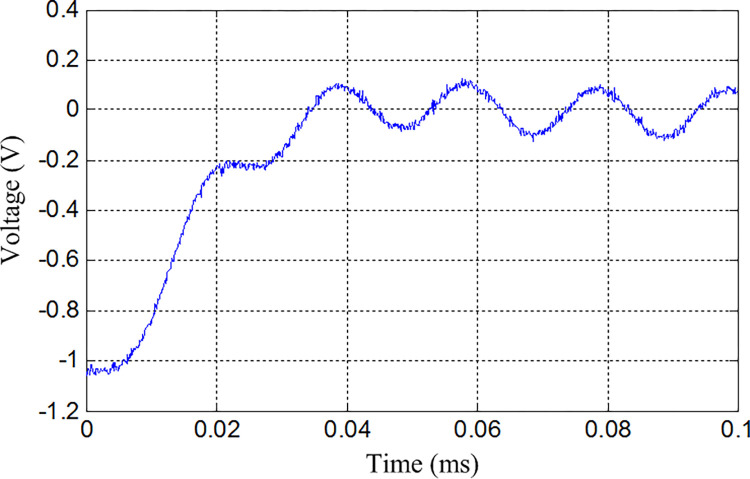
The output signal of the biquad filter circuit under normal state.

The simulation experiments is performed using MATLAB 7.0. The CPU clock speed of the computer is 2.3 GHz, and the main memory is 8 GB. The input signal are *u*_1_ = cos(2*π*×5×10^4^*t*) and*u*_2_ = 1.5 cos(2*π*×5×10^4^*t*), the sampling frequency is 1×10^7^ Hz, and the sampling length is 1×10^4^ s. When the filter circuit output is affected by the Gaussian white noise, and the signal-to-noise ratio is 40 dB, the output signal of the biquad filter circuit under normal state is shown in Fig 4.

The Monte Carlo method is used in the fault diagnosis simulation of the biquad filter circuit, and the signal-to-noise ratio are 40 dB and 38 dB respectively. Assuming that the variation ranges of the circuit element parameters were within 5%, 200 sets of fault feature samples are collected for each fault mode based on the GFRF.

**Fig 5:**
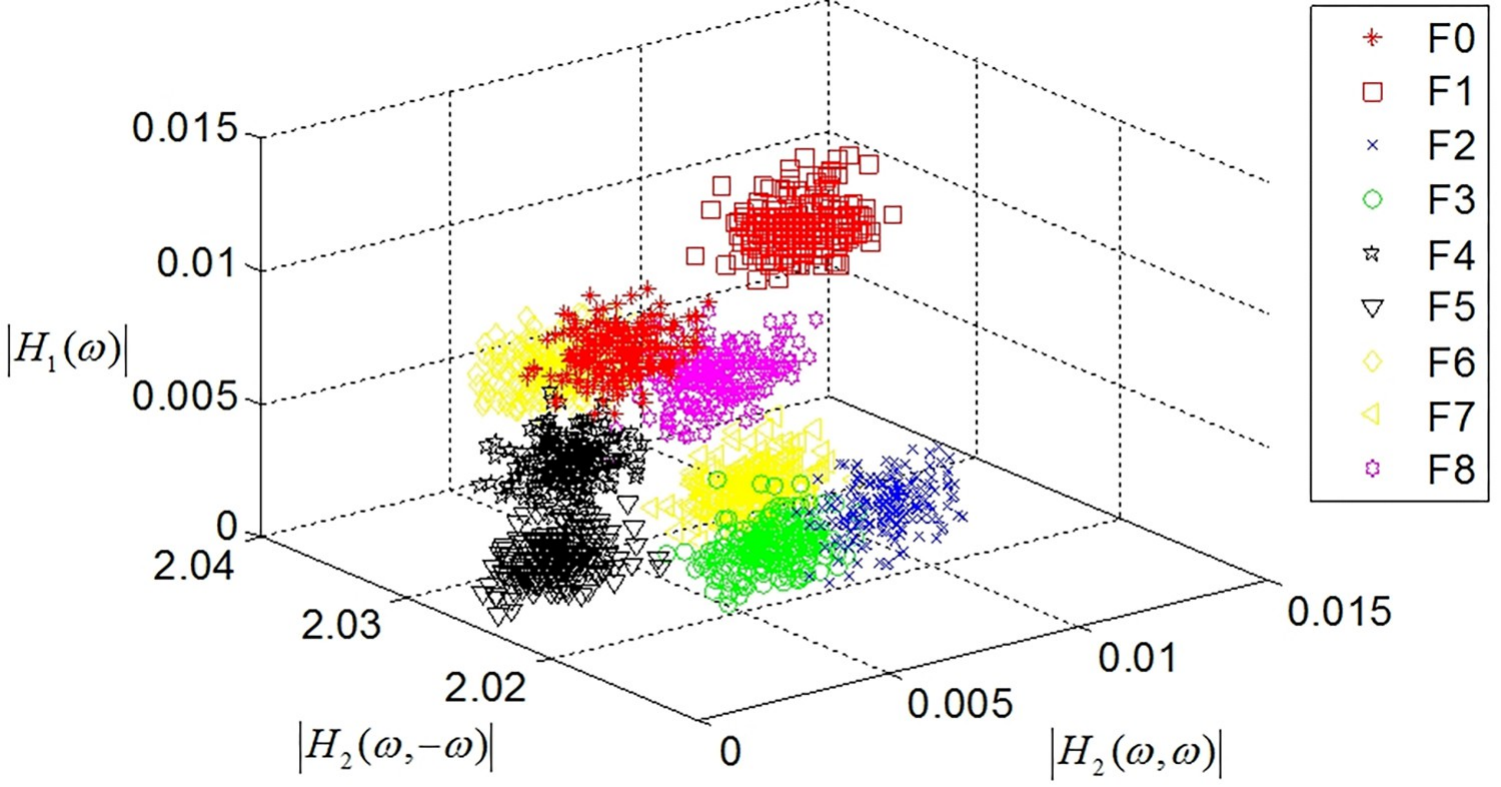
The amplitude distributions of the first- and second-order GFRFs.

**Fig 6:**
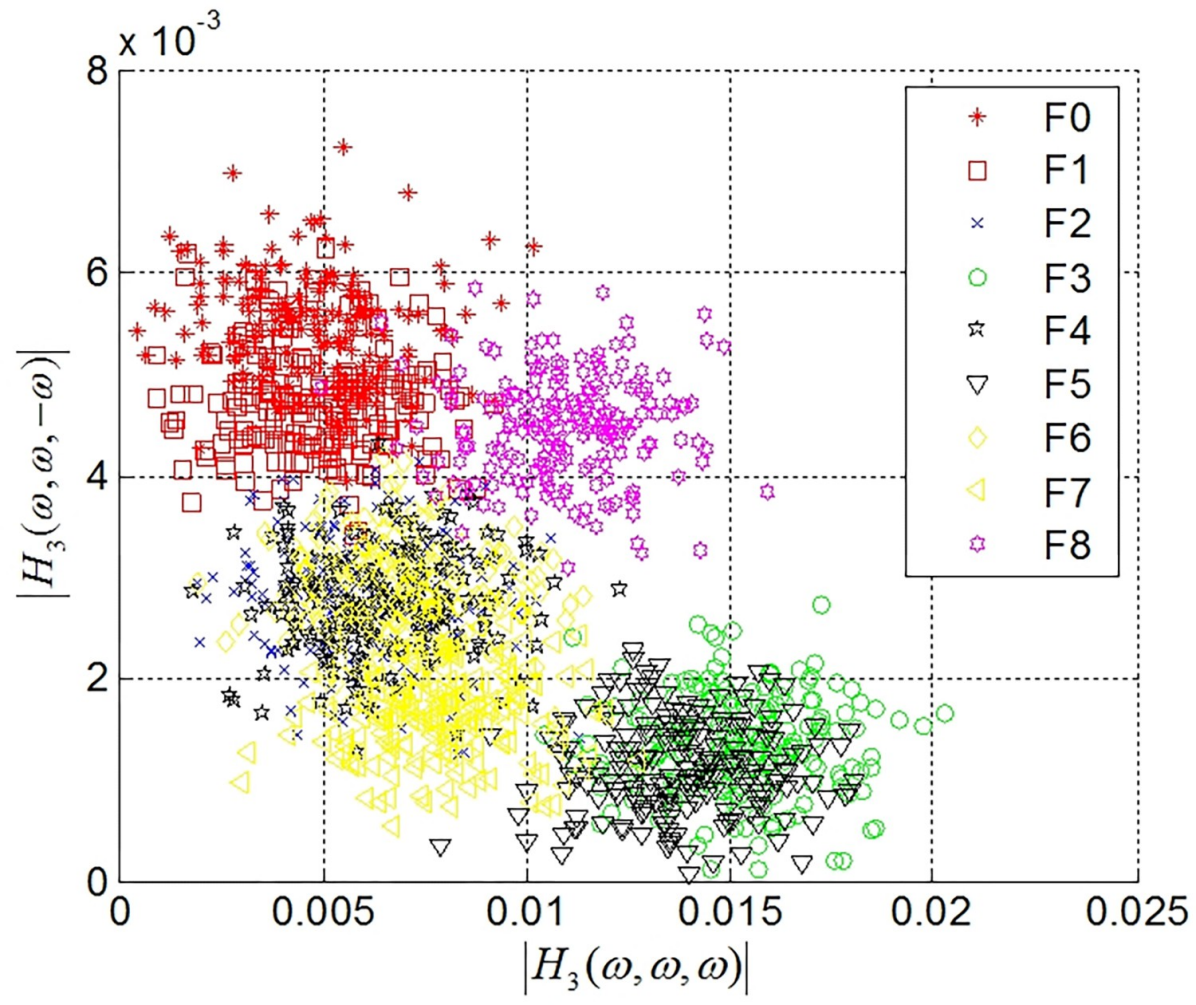
The amplitude distributions of the third-order GFRF.

The amplitude distributions of the estimated GFRF at 40 dB are shown in Figs 5 and 6, where Fig 5 shows the amplitude distribution of the first- and second-order GFRFs, and Fig 6 shows the amplitude distributions of the third-order GFRF. As shown in Figs 5 and 6, the amplitude distributions of the GFRF of nine fault modes are complex, and there are obvious intersections in the amplitude distribution regions of GFRF in different fault.

For each fault mode, 100 sets of the feature data are selected as training samples of the LSSVM multi-fault classifier. The remaining 100 sets of the feature data are used as test samples to verify the diagnosis performance of the proposed classifier. The Gaussian radial basis function is chosen as the LSSVM kernel function. The radial basis function width is *σ* = 8x10^6^, the penalty factor is *c* = 9×10^−4^.

The recognition rate of the proposed LSSVM multi-fault classifier for the biquad filter circuit is given in Table 2, where it can be seen that the designed multi-fault classifier achieved good diagnosis performance. The fault recognition rate is very high under both signal-to-noise ratios.

**Table 2:** The recognition rate of the multi fault classifier circuit.

Fault code	Fault description	Recognition rate	
		40 dB (SNR)	38 dB (SNR)
F0	NF	97%	100%
F1	R3↑	98%	92%
F2	R3↓	99%	95%
F3	R5↑	96%	89%
F4	R5↓	96%	90%
F5	C1↑	95%	82%
F6	C1↓	99%	95%
F7	C2↑	100%	92%
F8	C2↓	100%	95%

The training time of the simplified LSSVM model is compared with the traditional LSSVM model [34]. These two models are trained 100 times respectively. The average value of each training for different kernel functions is given in Table 3, where it can be seen that the training time of the simplified LSSVM model is significantly reduced.

**Table 3:** The average training time for different kernel functions.

Kernel function	Average training time (s)	
	Traditional LSSVM	Simplified LSSVM
Gaussian radial basis function	2.69	2.05
Polynomial function	3.58	3.43
Linear function	2.67	2.19

The proposed fault diagnosis method could be effectively used for fault diagnosis of analog circuits. In the future, the excitation signal optimization could be an important aspect of studying. By optimizing the excitation signal, GFRFs that more significantly reflect nonlinear circuit fault could be obtained.

## 5. Conclusions

Based on the GFRF and the simplified model of the least squares vector machine, this paper proposes an improved fault diagnosis method for a nonlinear analog circuit. Because the GFRF can effectively reflect the frequency characteristics of nonlinear systems, there can exist significant differences in the GFRFs of a nonlinear system under different fault modes. Therefore, the GFRFs of a nonlinear analog circuit are used to obtain fault features. After obtaining the fault features, the least squares support vector is used to construct a multi-fault classifier for fault recognition. To improve training speed and reduce storage, the conjugate gradient algorithm is combined with a simplified LSSVM model. The performance of the proposed method is verified by the fault diagnosis simulation experiment on a biquad filter circuit. The experimental results show that the proposed fault diagnosis method for nonlinear analog circuits has a high recognition rate and can significantly reduce the training time. The proposed fault diagnosis method can be effectively used for analog circuit fault diagnosis, providing a research area for circuit fault diagnosis.

The proposed diagnosis method for nonlinear analog circuits could be further improved. For instance, to obtain more GFRFs of a nonlinear analog circuit, a multi-tone signal could be used as an excitation signal. An important aspect of studying in the future could be the estimation of the GFRF under a multi-tone excitation signal, involving the estimation algorithm and excitation signal optimization. In addition, the structural design and sparsity of the proposed LSSVM multi-classifier could be studied more deeply.

## Supporting information

S1 Data (ZIP)

S2 Data (ZIP)

S3 Data (ZIP)

S4 Data (ZIP)

S5 Data (ZIP)

S1 Code (ZIP)
